# Ursolic Acid’s Alluring Journey: One Triterpenoid vs. Cancer Hallmarks

**DOI:** 10.3390/molecules28237897

**Published:** 2023-12-01

**Authors:** Youness Limami, Aline Pinon, Hicham Wahnou, Mounia Oudghiri, Bertrand Liagre, Alain Simon, Raphaël Emmanuel Duval

**Affiliations:** 1 Laboratory of Health Sciences and Technologies, Higher Institute of Health Sciences, Hassan First University of Settat, Settat 26000, Morocco; 2 Laboratory of Immunology and Biodiversity, Faculty of Sciences Ain Chock, Hassan II University, B.P. 2693, Maarif, Casablanca 20100, Morocco; hwwahnou@gmail.com (H.W.); mounia.oudghiri@univh2c.ma (M.O.); 3 Univ. Limoges, LABCiS, UR 22722, F-87000 Limoges, France; aline.pinon@unilim.fr (A.P.); bertrand.liagre@unilim.fr (B.L.);; 4 Université de Lorraine, CNRS, L2CM, F-54000 Nancy, France

**Keywords:** ursolic acid, cancer hallmarks, cell growth, apoptosis, angiogenesis, signaling pathways

## Abstract

Cancer is a multifactorial disease characterized by various hallmarks, including uncontrolled cell growth, evasion of apoptosis, sustained angiogenesis, tissue invasion, and metastasis, among others. Traditional cancer therapies often target specific hallmarks, leading to limited efficacy and the development of resistance. Thus, there is a growing need for alternative strategies that can address multiple hallmarks concomitantly. Ursolic acid (UA), a naturally occurring pentacyclic triterpenoid, has recently emerged as a promising candidate for multitargeted cancer therapy. This review aims to summarize the current knowledge on the anticancer properties of UA, focusing on its ability to modulate various cancer hallmarks. The literature reveals that UA exhibits potent anticancer effects through diverse mechanisms, including the inhibition of cell proliferation, induction of apoptosis, suppression of angiogenesis, inhibition of metastasis, and modulation of the tumor microenvironment. Additionally, UA has demonstrated promising activity against different cancer types (e.g., breast, lung, prostate, colon, and liver) by targeting various cancer hallmarks. This review discusses the molecular targets and signaling pathways involved in the anticancer effects of UA. Notably, UA has been found to modulate key signaling pathways, such as PI3K/Akt, MAPK/ERK, NF-κB, and Wnt/β-catenin, which play crucial roles in cancer development and progression. Moreover, the ability of UA to destroy cancer cells through various mechanisms (e.g., apoptosis, autophagy, inhibiting cell growth, dysregulating cancer cell metabolism, etc.) contributes to its multitargeted effects on cancer hallmarks. Despite promising anticancer effects, this review acknowledges hurdles related to UA’s low bioavailability, emphasizing the need for enhanced therapeutic strategies.

## 1. Introduction

**Figure 1:**
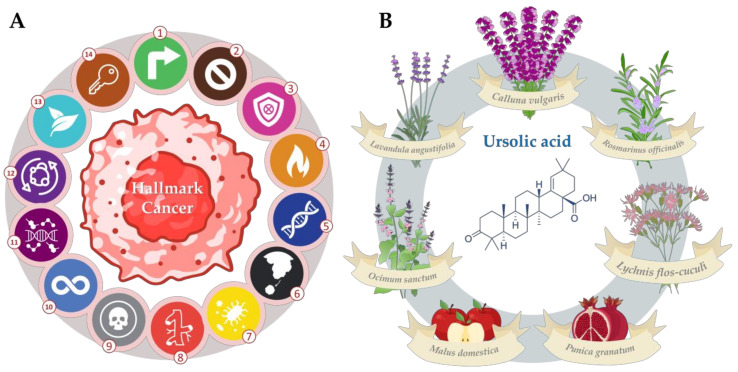
(**A**) The fourteen hallmarks of cancer [1,2,3] classified, respectively, as follows: (1) sustaining proliferative signaling, (2) evading growth suppressors, (3) avoiding immune destruction, (4) tumor-promoting inflammation, (5) genome instability and mutation, (6) activating invasion and metastasis, (7) polymorphic microbiomes, (8) inducing angiogenesis, (9) resisting cell death, (10) enabling replicative immortality, (11) non-mutational epigenetic reprogramming, (12) deregulating cellular energetics, (13) senescent cells, (14) unlocking phenotypic plasticity. (**B**) Some botanical sources of ursolic acid (UA), the structure of UA is represented at the center.

In the early 2000s, Hanahan and Weinberg introduced a conceptual framework highlighting distinct capabilities cells acquired during the progression toward a neoplastic state. These characteristics, collectively referred to as “hallmarks of cancer”, have significantly contributed to our understanding of tumor pathogenesis [1]. The original hallmarks of cancer encompassed pivotal features such as sustaining proliferative signaling, evading growth suppressors, resisting cell death, enabling replicative immortality, inducing angiogenesis, and activating invasion and metastasis. Genome instability and mutation profoundly influenced these hallmarks, which played a crucial role in their manifestation [1] (Figure 1A).

To account for advancements in scientific knowledge and incorporate new insights, Hanahan and Weinberg revised their framework in 2011 [2]. The updated version included additional hallmarks such as reprogramming of energy metabolism, avoidance of immune destruction, promotion of tumor-related inflammation, and evasion of immune surveillance. This expansion provided a more comprehensive understanding of the complex interplay between cancer cells and their microenvironment [2] (Figure 1A).

Building upon these advancements, Hanahan further refined the framework in 2022, introducing four emerging hallmarks and enabling characteristics including non-mutational epigenetic reprogramming, senescent cells, unlocking phenotypic plasticity, and polymorphic microbiomes [3]. These novel additions are poised to enhance our comprehension of the multifaceted nature of cancer (Figure 1A). The continuous evolution of this framework underscores the dynamic nature of cancer research and highlights the need for ongoing investigations to elucidate the underlying mechanisms driving cancer progression.

In this context, phytochemicals, especially ursolic acid (UA), have garnered significant attention as potential therapeutic agents. UA is a common secondary metabolite, belonging to ursane-type pentacyclic triterpenoids found in a large variety of medicinal herbs and plants [4,5] (Figure 1B). Prominent examples of plants containing significant amounts of ursolic acid include apple peels, rosemary, basil, oregano, thyme, and lavender [6]. In addition to fruits and herbs, certain medicinal plants such as holy basil (*Ocimum sanctum*) [7] and the Chinese herb known as “Loquat” (*Eriobotrya japonica*) also serve as rich sources of UA [8]. The compound is present in the waxy coatings of fruits and leaves, contributing to its protective role against environmental stressors. The diverse array of sources underscores the potential for harnessing ursolic acid from natural botanicals for various therapeutic and nutritional purposes.

UA can exhibit pleiotropic effects, acting on multiple targets simultaneously, and often possesses the ability to influence several hallmarks of cancer (Table 1). In fact, UA can impact cell proliferation, apoptosis, angiogenesis, metastasis, and inflammation, among other processes. It can influence key players within these pathways, including transcription factors, enzymes, cell cycle regulators, and receptor tyrosine kinases. Importantly, the multitargeted nature of UA aligns with the concept of simultaneously targeting multiple hallmarks, which is essential for effective cancer therapy [1,2,3].

In tandem with the exploration of UA potential in targeting cancer hallmarks, it is crucial to appreciate the broader context of cancer research and treatment. Cancer, a complex and multifaceted disease, arises from various factors contributing to its etiology, including genetic predispositions, environmental exposures, and lifestyle choices [9]. The diagnosis of cancer involves sophisticated techniques, such as imaging studies, biopsies, and molecular analyses, to ascertain the nature and extent of the disease [10]. Current cancer therapies encompass a spectrum of approaches, including surgery, chemotherapy, radiation therapy, immunotherapy, and targeted therapies [10]. Each modality carries its unique set of benefits and challenges, often accompanied by side effects that can impact patients’ quality of life. Recognizing the nuanced landscape of cancer etiology, diagnostics, and therapies provides a comprehensive backdrop for evaluating the potential of UA as an adjunct or complementary agent in the intricate tapestry of cancer treatment.

The evolving understanding of cancer hallmarks, the identification of emerging hallmarks, and the recognition of the multifaceted nature of cancer progression underscore the necessity for ongoing investigations. Elucidating the underlying mechanisms and identifying new targets within signaling pathways are crucial for the development of targeted and effective therapeutic interventions. Moreover, exploring the potential of phytochemicals, such as UA, in modulating these pathways and targets opens up exciting avenues for the discovery of novel anti-cancer strategies. This review aims to explore the potential of UA, in targeting and modulating cancer hallmarks. The review will shed light on the molecular targets and signaling pathways through which UA exerts its multitargeted effects on cancer hallmarks. By providing a comprehensive analysis, this review seeks to contribute to the development of effective strategies for combating cancer through multitargeted approaches. Additionally, the manuscript delves into the challenges associated with UA research, including issues of low bioavailability, the necessity for more extensive in vivo studies, and the variability in doses used in different experimental settings. Furthermore, innovative perspectives, such as the potential of synthetic UA and nanoformulation strategies, are discussed, offering insights into overcoming these challenges and paving the way for the advancement of UA-based cancer therapies.

To ensure the precision and comprehensiveness of our review, our team meticulously conducted a thorough data collection and search across reputable databases, including Pubmed, Google Scholar, Springer, Elsevier ScienceDirect, and Web of Science. Our initial focus was on gathering insights into the targets of cancer hallmarks as elucidated in the renowned reviews by Hanahan and Weinberg [1,2,3]. Subsequently, we systematically evaluated UA’s potential to target these hallmarks through keyword and heading searches, employing terms such as “cancer chemoprevention”, “anti-tumor”, “anti-cancer”, “cell death”, “cell cycle arrest”, “in vivo studies”, and “in vitro studies”. Furthermore, to maintain consistency and ensure a comprehensive analysis, we utilized the main pathways and markers delineated in Table 1 as additional keywords. Our scrutiny extended to studies published between 2000 and 2023. 

Implementing stringent inclusion criteria, we selected original articles and review papers that met specific benchmarks, assuring the precision and quality of the information presented in this paper. In total, 76 references were thoughtfully included in our study.

## 2. UA Targets Cancer Hallmarks

Extensive research has led scientists to uncover numerous cancer hallmarks and pathways that serve as potential targets for therapeutic interventions. These hallmarks encompass a wide range of factors, including proteins, signaling pathways, cellular processes, and epigenetic modifications, as outlined in Table 1. By specifically targeting these hallmarks, there is a significant opportunity to develop personalized and highly effective cancer therapies. In the subsequent sections, we will delve into each cancer hallmark, elucidating the principal pathways and markers involved, and explore how UA can effectively affect them.

### 2.1. Hallmark 1: Inhibition of Proliferative Signaling

Normal cells rely on tightly regulated cell cycle control mechanisms to ensure controlled proliferation and maintain the equilibrium of tissues. However, this precise control system is disrupted in cancer. Unlike normal cells, cancer cells exhibit a unique characteristic of releasing and responding to growth factors, particularly epidermal growth factor (EGF) and its receptor (EGFR) signaling [11]. By activating these growth factors, cancer cells acquire a heightened ability to stimulate their growth and division, independent of external signals. This phenomenon, known as self-sufficiency in cell proliferation, is primarily driven by three major signaling pathways, Akt, MAPK/ERK, and mTOR [2]. These pathways act as crucial drivers, fueling the uncontrolled growth and replication of cancer cells. Their activation promotes the survival and expansion of cancer cells, contributing to the relentless progression of the disease.

**Figure 2:**
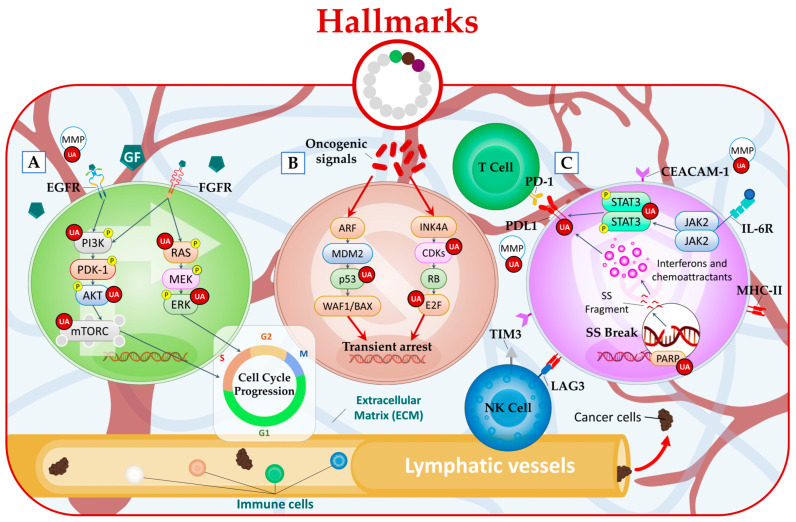
UA targets cancer by (**A**) inhibiting of proliferative signaling, (**B**) inhibiting of growth suppressors, and (**C**) inhibiting of immune evasion.

It has been revealed that UA exhibits multifaceted effects on various signaling pathways involved in cell cycle progression. UA has shown a capacity to inhibit the phosphorylation/activation of Akt [11,12], mTOR [13], Ras [14], and ERK [15] in a dose-dependent manner (Figure 2A). Furthermore, Yang et al. examined the effects of UA treatment on NSCLC and H1975 lung cancer cell lines that carry the EGFR T790M mutation [16]. This mutation is a major cause of resistance to EGFR tyrosine kinase inhibitors (EGFR-TKIs) such as erlotinib [17]. The results demonstrated that UA treatment effectively suppressed cell proliferation and motility while inducing apoptosis in these cells [16]. Furthermore, the study also extended its investigation to animal models by xenografting lung cancer cells that expressed the EGFR T790M mutation, providing additional evidence supporting the potential efficacy of UA in treating this specific subtype of NSCLC [16]. In a related context, UA markedly deterred the growth of xenograft tumors in colorectal cancer (CRC) models. The in vivo experiments revealed that UA not only ameliorated pathological features but also triggered apoptosis and arrested the cell cycle in xenograft CRC tissue [18]. These effects were attributed to the downregulation of the Wnt/β-catenin signaling cascade [18]. The comprehensive findings from both cellular and animal model studies underscore the potential of UA as a versatile therapeutic agent with promising anti-cancer properties, supporting its further exploration for the treatment of various cancer subtypes, including NSCLC and CRC.

### 2.2. Hallmark 2: Inhibition of Growth Suppressors

To bypass the growth-inhibitory signals originating from normal homeostatic processes, cancer cells exhibit a reduced responsiveness to external signals that normally impede their growth. These cells actively resist apoptotic control, which is crucial for tightly regulating cell death and curbing uncontrolled proliferation [2].

In normal conditions, apoptosis plays a pivotal role in eliminating cells that undergo excessive proliferation, thus maintaining a balanced cell population and removing aberrant or diseased cells [19]. On the other hand, autophagy serves as a cellular recycling system, responsible for eliminating abnormal proteins and cellular components, while promoting cellular regeneration. Cancer cells develop resistance to apoptotic signals, preventing programmed cell death and enhancing their survival [19]. Furthermore, cancer cells manipulate autophagy to enhance their growth potential and overcome nutrient scarcity, facilitating their survival and uncontrolled proliferation, even in challenging conditions [2].

This aberrant cellular behavior is primarily driven by Rb and p53 proteins, two prototypical tumor suppressors [13]. Interestingly, emerging research suggests that UA holds promise in targeting both proteins. Studies using human non-small cell lung cancer A549 cells showed the ability of UA to induce G1-phase arrest produced through the p53/p21-mediated and Cdks-inhibition pathway [20]. UA showed also an ability to increase the expression of E2F, a protein involved in the Rb signaling pathway (Figure 2B) [21]. 

Nevertheless, p53 and Rb operate within a larger network with functional redundancy, demonstrating that multiple mechanisms regulate cell proliferation [22]. This complex interplay ensures robust control over cell growth even in the absence of p53 or Rb (Table 1).

### 2.3. Hallmark 3: Inhibition of Immune Evasion

The human immune system is a complex network of cells, tissues, and organs that plays a crucial role in defending the body against foreign pathogens and diseases [23]. However, its importance extends beyond infection control, as it also functions to identify and eliminate unhealthy and ailing cells, including cancer cells [23]. T cells and NK cells are crucial in the fight against cancer [17]. T cells recognize tumor antigens and orchestrate immune responses, while NK cells detect and eliminate cancer cells directly [23].

In cancer immunotherapy, targeting immune checkpoint molecules like PD1/PD-L1, TIM3, and LAG3 has revolutionized treatment approaches [2]. These molecules regulate the activity of T cells, and blocking them can unleash the immune system’s ability to recognize and attack cancer cells more effectively [23]. UA has shown promise in targeting some proteins involved in the immune evasion of cancer. Kang et al. showed that UA can exhibit anticancer activities by inhibiting MMP2 and PD-L1 expression through EGFR/JAK2/STAT3 signaling in NSCLC cells A549 and H460 (Figure 2C) [24,25].

### 2.4. Hallmark 4: Reducing Tumor Inflammation

The tumor microenvironment manipulates immune cells to support tumor survival instead of eliminating cancer cells. Immune cells in the TME secrete chemicals such as reactive oxygen species (ROS) that are actively mutagenic for nearby cancer cells [26]. Immune cells also release factors that aid in tumor growth and metastasis, rather than identifying and destroying cancer cells [2,27]. Key inflammatory mechanisms affected by the tumor are NF-κB, immune checkpoint signaling, and inflammasome signaling [2].

**Figure 3:**
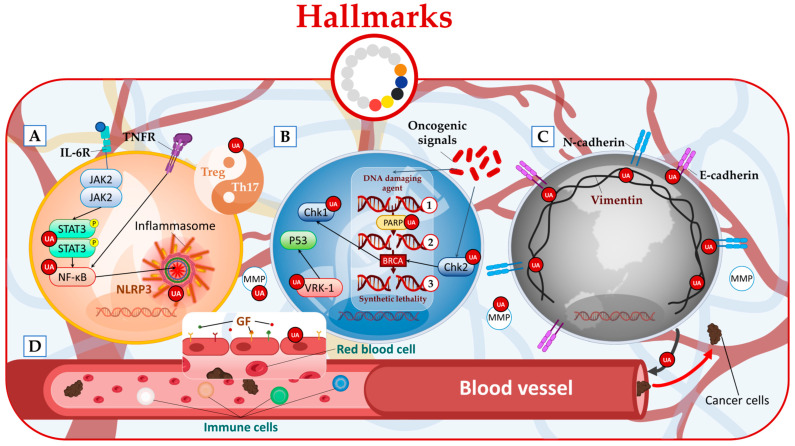
UA targets cancer by (**A**) reducing tumor inflammation, (**B**) regulating genome instability and mutation (where 1, 2, and 3 refer, respectively, to single-strand break, double-strand break, and DNA not repaired), (**C**) inhibiting cancer invasion metastasis, and (**D**) inhibiting angiogenesis.

One potential approach to counteract the tumor-mediated hijacking of immune cells in the tumor microenvironment is the use of UA. Studies have demonstrated that UA possesses the ability to reduce the levels of inflammatory proteins such as TNF-α, NF-κB, and cyclooxygenase-2 (COX-2) in several cancer cell lines and other inflammatory models mainly by targeting JAK/STAT signaling pathway [28,29,30,31,32,33]. Furthermore, UA can also act by inhibiting NLRP3 inflammasome activation (Figure 3A) [34].

By targeting these inflammatory pathways, UA can potentially alleviate the detrimental effects of chronic inflammation that can lead to carcinogenesis and promote cellular health.

### 2.5. Hallmark 5: Genome Instability and Mutation

Cancer cells exhibit a high rate of proliferation, which increases the likelihood of genetic alterations and mutations affecting genes involved in cell division and tumor suppression. This genetic instability fosters the development of further cancerous adaptations within the cells. These changes can arise from direct DNA mutations or epigenetic modifications, which can disrupt protein expression levels and compromise genomic integrity [2,3]. Precision cancer therapies have been developed to target specific components of the cell cycle, such as checkpoint kinases (e.g., Chk1 and Chk2 proteins), as well as DNA damage repair enzymes like BRCA and 53BP1. These therapies aim to disrupt the molecular mechanisms that facilitate cancer cell proliferation and survival, offering potential avenues for more precise and effective treatments [3,35].

In the same context, it has been observed that UA exhibits the capacity to effectively inhibit the phosphorylation of Chk1, Chk2, and BRCA in a dose-dependent manner [36], as illustrated in Figure 3B. Furthermore, the administration of UA has effectively modulated the generation of both cellular and mitochondrial ROS. This, in turn, triggers a response in embryonic CSCs known as DNA damage response (DDR), strongly suggesting the potential for UA-induced cell death [36].

UA also weakens surveillance mechanisms by blocking 53BP1 foci formation induced by VRK1. This is achieved by inhibiting the catalytic activity of VRK1 through direct binding to its catalytic domain [35].

### 2.6. Hallmark 6: Activating Invasion and Metastasis

Tissue invasion refers to the process by which tumor cells expand into nearby tissues, while metastasis involves the migration of tumor cells from the primary tumor site to distant locations, where they establish secondary tumors [1,37]. One key mechanism involved in these processes is the well-documented epithelial-to-mesenchymal transition, which plays a crucial role in facilitating uninhibited cell division and enabling metabolic adaptations that promote cell survival under nutrient-deprived and stressful conditions [1,2,37].

These cancer mechanisms entail extensive modifications in cell–cell and cell–matrix interactions, as well as cellular transformations that promote invasion and migration. Specific targets involved in these processes include collagen and CEACAM1, which undergo alterations to facilitate the behavior of cancer cells [2,24,37]. These changes contribute to the invasive properties of cancer cells, allowing them to penetrate tissues and migrate to distant sites, ultimately leading to the establishment of secondary tumors [37].

In studies conducted on mesothelioma cells, it has been observed that UA exhibits the ability to impede the process of epithelial-to-mesenchymal transition (EMT). Specifically, UA activates E-cadherin, while concurrently inhibiting the expression of N-cadherin, vimentin, and Twist (Figure 3C) [12]. In addition, studies involving 4T1 tumor-bearing mice have shown that UA can inhibit lung metastasis [21]. Furthermore, UA also inhibits MMP2 an enzyme that facilitates the breakdown of the extracellular matrix, allowing cancer cells to invade surrounding tissues and enter blood vessels or lymphatics for metastatic dissemination [24].

### 2.7. Hallmark 7: Polymorphic Microbiomes

Variations in the microbial communities within our bodies, known as microbiomes, have been found to have a significant impact on cancer characteristics, development, and progression. Research suggests that polymorphic microbiomes can serve as a distinctive enabling factor that influences the acquisition of cancer hallmarks, potentially promoting or protecting against different types of cancer [38,39]. For instance, studies have revealed the presence of microbiomes with either cancer-promoting or cancer-protective properties, influencing the incidence and progression of colon cancer [39]. Furthermore, the composition of the gut microbiome has been shown to influence the immune system, affecting anti-tumor immunity and the response to immunotherapy in patients with melanoma [40,41]. Notably, the emerging concept of polymorphic microbiomes intersects with established hallmarks such as genome instability, mutations, and tumor-promoting inflammation [3]. This indicates that the composition of microbiomes can significantly contribute to the development and progression of cancer, acting alongside other well-known cancer hallmarks.

UA has shown potential in modulating the composition of the gut microbiota and improving the microenvironment within the digestive system [42]. Studies have demonstrated that UA can bring about changes in the gut microbiota by promoting the growth and enrichment of beneficial bacteria such as *Bifidobacterium* spp and *Lactobacillus* spp [42,43]. These alterations in the microbial composition contribute to a healthier gut environment. Moreover, the presence of an enhanced population of *Bifidobacterium* spp and *Lactobacillus* spp has been associated with immune regulatory effects [43]. Additionally, UA’s ability to promote the growth of these beneficial bacteria was correlated with its ability to correct the imbalance of Th17/Treg cells (Figure 3A) [43].

### 2.8. Hallmark 8: Reducing Angiogenesis

The establishment and growth of the vascular network play a pivotal role in metastasis as cancer cells require a sufficient supply of nutrients and oxygen, as well as a means of waste removal [44]. This crucial process is achieved through two mechanisms: angiogenesis, which involves the formation of new blood vessels, and lymphangiogenesis, which involves the formation of lymphatic vessels [44]. Abnormal signaling of specific growth factors, such as VEGF, FGF-β, and PDGF, significantly contribute to the promotion of tumor angiogenesis [1,2,44]. These growth factors play a significant role in stimulating the formation of blood vessels within tumors.

In vivo and in vitro studies conducted by Lin et al. using mice colorectal cancer (CRC) xenograft have shown that UA significantly reduced the intratumoral microvessel density [45]. Furthermore, by (i) suppressing the proliferation of endothelial cells, (ii) inhibiting their migration, and (iii) impeding the formation of capillary tubes, UA exhibits notable anti-angiogenic properties. Additionally, UA treatment significantly reduced the expression of VEGF-A and FGF-β in both CRC tumors and HT-29 cells (Figure 3D) [45].

### 2.9. Hallmarks 9 and 10: Resisting Cell Death and Enabling Replicative Immortality

Cancer cells possess mechanisms that enable them to resist cell death, a hallmark characteristic of cancer. This resistance is not solely due to a lack of response to external signals but rather arises from intrinsic mechanisms within the cancer cells. These mechanisms can be attributed to mutations that disrupt apoptotic signaling pathways or impair the detection of cellular damage [2,3].

Apoptosis, also known as programmed cell death, exhibits distinct features including cell shrinkage, membrane blebbing, chromosome condensation (pyknosis), nuclear fragmentation (karyorrhexis), DNA fragmentation, and eventual engulfment of the cell by phagocytes [46]. On the other hand, autophagy, another cellular process, plays a vital role in enabling cells to survive in the face of various stressful conditions [47]. Cancer cells exploit autophagy to overcome nutrient limitations and facilitate tumor growth [48]. Moreover, autophagy can impact the tumor microenvironment by promoting angiogenesis, providing nutrients, and modulating inflammatory responses [2,19].

Cancer cells often exhibit alterations in key apoptotic signaling proteins such as caspases, Bcl-2 family, and p53. These proteins may be downregulated, mutated, or bypassed, contributing to the resistance of cancer cells to apoptosis. Similarly, the regulation of autophagy in cancer involves targeting specific pathways, including MAPK, ATG, and p62, which are involved in autophagy-related processes [2,19].

**Figure 4:**
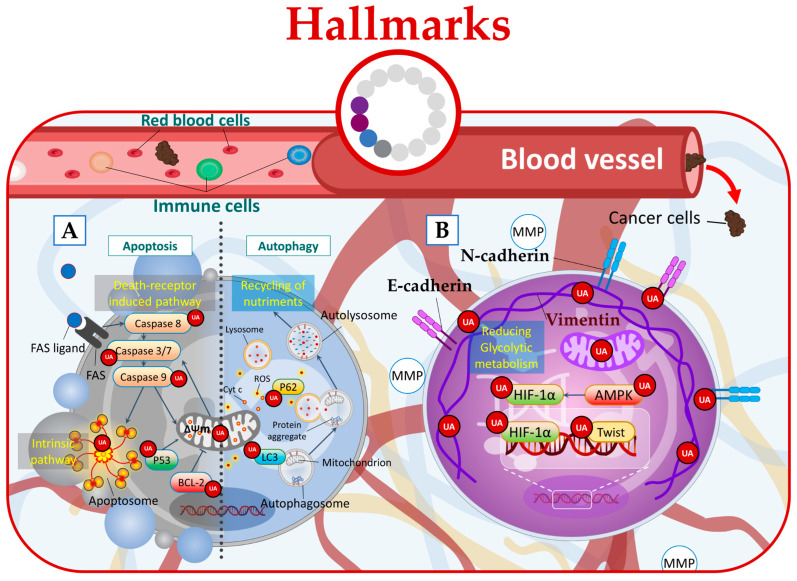
UA targets cancer by (**A**) inducing apoptosis and autophagy, (**B**) regulating microenvironmental factors, and disrupting cancer energy metabolism.

Notably, UA has been shown to elicit distinct effects on either mitochondrial or death receptors apoptotic pathways including the upregulation of the Fas receptor the cleavage of caspases-8, -3, -7, and -9, the regulation of Bcl-2 family proteins, and the activation of p53 leading to apoptotic cell death (Figure 4A) [15,28,49,50,51,52,53,54].

Furthermore, in phenotypically breast cancer cells, UA has demonstrated its anticancer potential by targeting glycolysis. By inhibiting Akt and inducing changes in the glycolytic pathway, UA leads to energy stress and activates AMPK (adenosine monophosphate-activated protein kinase), ultimately promoting a combined effect of autophagy and apoptosis at low micromolar concentrations (Figure 4A) [55].

Additionally, UA treatment was found to induce autophagy in Esophageal Squamous Cell Carcinoma (ESCC) cells. This was evidenced by the increased levels of LC3-II protein, which is a marker of autophagosome formation, and the concurrent decrease in p62 protein levels [56]. Moreover, UA increased cellular ROS leading to ROS-dependent autophagy (Figure 4A) [56].

### 2.10. Hallmark 11: Non-Mutational Epigenetic Reprogramming

Non-mutational epigenetic reprogramming can occur through microenvironmental factors such as hypoxia or EMT [57]. Hypoxia, for instance, can induce hypermethylation by suppressing the activity of TET demethylases. EMT is responsible for the reversible induction of cancer cell invasiveness at the periphery of solid tumors. Epigenetic regulatory heterogeneity is a significant aspect of non-mutational epigenetic reprogramming [3].

In this context, UA emerges as a potential therapeutic agent capable of targeting the hypoxic tumor microenvironment directly. It achieves this by downregulating HIF-1α (hypoxia-inducible factor-1α), an oxygen-dependent transcriptional activator [21], or through AMPK modulation [58]. By targeting these key regulators, UA has the potential to disrupt the hypoxic microenvironment of tumors. Additionally, UA exhibits the ability to target EMT by acting on crucial pathways and markers involved, including TGF-β, Wnt [59], SNAIL, Twist, cadherins, and vimentin (Figure 4B) [60].

### 2.11. Hallmark 12: Deregulating Cellular Energetics

Due to their rapid proliferation, cancer cells have heightened energy and nutrient demands, even in environments with limited oxygen supply [3]. This necessitates significant metabolic alterations in cancer cells to meet their metabolic needs. One prominent metabolic adaptation observed in cancer cells is the Warburg effect. This phenomenon involves a shift towards increased glycolysis, even in the presence of oxygen, leading to the diversion of pyruvate away from the Krebs cycle and towards lactate production [3,61]. This altered metabolic pathway allows cancer cells to generate energy quickly, although less efficiently than oxidative phosphorylation. In addition to the reliance on glycolysis, cancer cells often exhibit an increased utilization of glutamine to support their rapid proliferation [3].

Targeting the hypoxic tumor microenvironment involves focusing on key regulators such as HIF-1α and AMPK. HIF-1α plays a vital role in enabling cancer cells to adapt to hypoxia by promoting the expression of genes involved in glycolysis, angiogenesis, and cell survival. AMPK, on the other hand, is a metabolic sensor that switches to a tumor-promoting mode to protect against metabolic, oxidative, and genotoxic stress. Modulating these factors presents potential therapeutic avenues to disrupt the tumor microenvironment and interfere with cancer cell metabolism [3].

In this regard, UA demonstrates its potential by effectively targeting hypoxia through the HIF-1α pathway, leading to the downregulation of HIF-1α protein expression [21]. Furthermore, UA can disrupt the energy metabolism of cancer cells by directly acting on the glycolytic metabolism and mitochondrial respiration function [61]. Additionally, UA derivatives showed the ability to suppress cancer cell glucose metabolism by acting on 2-deoxy-D-glucose (2-DG) (Figure 4B) [57].

### 2.12. Hallmark 13: Senescent Cells

Cellular senescence is a process of irreversible cell cycle arrest that likely evolved as a protective mechanism to maintain tissue homeostasis [62]. It involves the cessation of cell division, accompanied by changes in cell morphology, metabolism, and the activation of senescence-associated secretory phenotype (SASP) [3,62]. Various internal and external factors, such as nutrient deprivation, DNA damage, organelle dysfunction, and disruptions in cellular signaling, can trigger senescence [62]. While senescent cells typically function as a defense against neoplasia, it is important to note that in certain cases, they can paradoxically promote tumor development and progression [63]. The secreted cytokines and growth factors from senescent cells, known as the SASP, can have either tumor-suppressive or oncogenic effects depending on the specific cellular context, cell type, and characteristics of the tumor [63]. In a recent review, Hanahan suggests considering senescent cells as functionally significant components of the tumor microenvironment [3].

Senescence-associated beta-galactosidase and uPAR (urokinase plasminogen activator receptor) are commonly used markers to identify senescent cells, with uPAR being a more recently described biomarker that exhibits broad induction during senescence.

**Figure 5:**
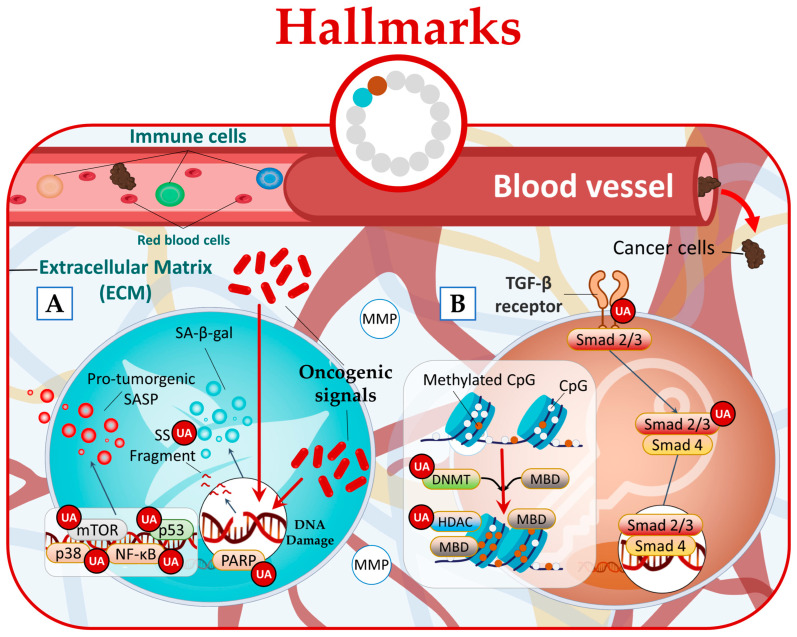
UA targets cancer by (**A**) reducing senescence state and (**B**) disrupting phenotypic plasticity.

In a cancer context, there is limited research investigating the impact of natural compounds on senescent cells. However, a study conducted by Han et al. shed light on this subject and revealed that UA effectively reduced senescence-associated beta-galactosidase (SA-β-gal) activity [64]. Furthermore, signaling pathways that regulate SASP, such as NF-κB, mTOR, and p38, or individual SASP factors that promote tumor progression [63] can also be targeted by UA (Figure 5A) [13,29,65].

### 2.13. Hallmark 14: Unlocking Phenotypic Plasticity

Cancer cells possess a unique ability to exhibit phenotypic plasticity, a characteristic that is typically restricted in normal cells. This plasticity plays a critical role in cancer initiation and progression by enabling disrupted differentiation states. It is considered a significant component of cancer pathogenesis [3]. It proposed that phenotypic plasticity represents a distinct capability within cancer, separate from the established core hallmarks.

Within this context, they identify three primary types of phenotypic plasticity. The first type involves the dedifferentiation of mature cells, which involves the reversion of these cells back to progenitor states. In colon cancer, for instance, the loss of transcription factors HOXA5 and SMAD4 is observed in advanced colon carcinomas, whereas they are highly expressed in differentiating epithelial cells. The second type encompasses blocked differentiation, where cancer cells are trapped in progenitor or stem cell states. This phenomenon is observed in various cancers such as acute promyelocytic leukemia (involving retinoic acid α nuclear receptor—RARα), acute myeloid leukemia, melanoma, and pancreatic cancer. The third type, known as transdifferentiation, occurs in pancreatic ductal adenocarcinoma (PDAC), where pancreatic acinar cells transform into a ductal cell phenotype. This process is regulated by transcription factors PTF1A and MIST1 [3].

Research studies have provided evidence that UA can disrupt all three forms of phenotypic plasticity. Specifically, UA has been found to effectively inhibit hyperproliferation of human dermal fibroblasts by regulating the Smad2/3 pathway [66]. Furthermore, UA has been found to possess the ability to downregulate the expression of epigenetic-modifying enzymes. This includes the DNA methyltransferases (DNMTs) DNMT1 and DNMT3a, as well as the histone deacetylases (HDACs) HDAC1, HDAC2, HDAC3, and HDAC8 belonging to class I, and HDAC6 and HDAC7 belonging to class II (Figure 5B) [67,68,69].

## 3. Challenges and Considerations in UA Research

UA holds immense promise as a potential therapeutic agent, demonstrating a range of beneficial effects in various in vitro studies. However, as we delve into the complexities of translating these findings into clinical applications, several challenges and considerations emerge. One significant hurdle lies in the need for more extensive in vivo studies to bridge the gap between laboratory findings and real-world applications. While in vitro studies provide valuable insights into the cellular mechanisms influenced by UA, the dynamic and intricate nature of biological systems necessitates a more comprehensive understanding through in vivo investigations [70]. Addressing this gap is crucial to establish the relevance and efficacy of UA in complex physiological environments.

Furthermore, the limited progress in clinical trials involving UA raises questions about its translational potential. A likely contributor to this shortfall is the well-documented issue of UA’s low bioavailability mainly because of its poor aqueous solubility/dissolution, poor permeability, and metabolism by cytochrome P450 (CYP) isozymes [71]. The challenges associated with ensuring effective delivery of UA to target tissues in vivo pose a considerable obstacle in achieving therapeutic concentrations. As we explore the multifaceted aspects of UA’s pharmacokinetics, it becomes evident that strategies to enhance bioavailability must be a focal point of future research efforts. Overcoming this hurdle could unlock the true therapeutic potential of UA and pave the way for its successful integration into clinical practice.

Additionally, a critical consideration in UA research is the substantial variance between doses used in various in vitro studies and physiologically achievable doses in vivo. Many in vitro investigations employ high concentrations of UA to elicit observable effects, yet these doses may not be feasibly replicated in living organisms. The challenge lies in deciphering the dose–response relationship and determining the optimal concentration range that can be safely administered in vivo. Striking this balance is pivotal to ensure that the therapeutic benefits observed in vitro can be translated into relevant and achievable outcomes in clinical settings.

## 4. Conclusions and Perspectives

In conclusion, UA shows great promise as a multi-targeted therapeutic agent for cancer treatment. Its ability to modulate various signaling pathways in vitro such as PI3K/Akt, MAPK/ERK, NF-κB, Wnt/β-catenin, and Smad2/3, among others, and to target all the fourteen hallmarks of cancer identified by Hanahan and Weinberg offers potential advantages over traditional therapies that target specific pathways and conventional treatment that have multiple complications [10,72,73]. This unique property positions UA as a promising lead candidate for the discovery of designed multiple ligands (DML) by engaging with multiple targets simultaneously. Nevertheless, the low bioavailability and targeting ability of the UA molecules remain challenging [71]. Hopefully, these challenges can be overcome through designing and developing synthetic UA by modifying its parent skeleton. Structural modifications offer a promising avenue to enhance the pharmacokinetic properties and target specificity of UA. By strategically introducing functional groups or altering specific moieties, researchers can tailor the synthetic derivatives to improve solubility, increase stability, and optimize interactions with target biomolecules [74].

Moreover, advancements in nanotechnology and drug delivery systems provide additional opportunities to address the bioavailability issue associated with UA [75]. The development of UA nanoformulations (e.g., micelles, liposomes, nanoemulsions, etc.) provides a strategy to overcome limitations such as poor gastrointestinal permeability and low oral absorption rate, enhancing drug delivery efficiency to the target site [75,76]. Future research in these areas should focus on optimizing UA nanoformulations, exploring combination therapies, and conducting clinical trials to fully realize their potential in improving cancer treatment outcomes.

## Figures and Tables

**Table 1:** Cancer hallmarks, mechanisms of action, and the main pathways and markers involved [1,2,3].

N°	Cancer Hallmark	Mechanisms of Action	Main Pathways/Markers	References
1	Sustaining proliferative signaling	Cellular proliferation	Cdk, Akt, MAPK/ERK, and mTOR	[1]
2	Evading growth suppressors	Tumor suppressors	Rb, p53	[1]
3	Avoiding immune destruction	Immune checkpoints	PD1/PD-L1, TIM3, MMP2, and LAG3	[2]
4	Tumor promoting inflammation	NF-κB signaling	NF-κB, IKK-β	[2]
		Tumor-associated macrophages	CD68, CD163, iNOS	[2]
5	Genome instability and mutation	Chromosomal instability	PARP, BRCA, 53BP1, and cyclin-dependent kinase	[2]
6	Activating invasion and metastasis	Extracellular matrix (ECM)	Hyaluronan, Versican, Collagen IV	[1]
		Adhesion molecules	CEACAM1, DCC, E-Cadherin	[1]
		Secreted factors	Tenascin C, Fibrinogen, Periostin	[1]
7	Polymorphic microbiomes	Gut dysbiosis	Microbiota	[3]
8	Inducing angiogenesis	Angiogenesis	VEGF, FGF-β, PDGF	[1]
9	Resisting cell death	Apoptosis	Caspases, Bcl-2, and p53	[1]
		Autophagy	MAPK, ATG, and p62	[1]
10	Enabling replicative immortality	Telomere regulation	TRF1/TRF2/POT1/TIN2/RAP1/TPP1	[1]
		p53 signaling	p53, MDM2, p14ARF/p19ARF, E2F-1	[1]
11	Non-mutational epigenetic reprogramming	Epithelial-to-mesenchymal transition (EMT)	TFG-β, Wnt, SNAIL, Twist, Cadherins, Vimentin	[3]
		Hypoxia	HIF1α/2α, HIF1β, CAIX, AP-1/c-jun, GLUT-1	[2,3]
12	Deregulating cellular energetics			
		Glycolysis	Tomm20, V-ATPase, GAPDH	[2]
		Mitochondrial metabolism	COX IV, VDAC1/Porin, ATPase β	[2]
13	Senescent Cells	Senescence-associated secretory phenotype	Senescence-associated β-galactosidase and uPAR	[3]
14	Unlocking phenotypic plasticity	Blocked differentiation	RAR α, HDAC, SOX10, α-ketoglutarate	[3]
		Dedifferentiation plasticity	HOXA5, SMAD4	[3]
		Transdifferentiation	PTF1A, MIST1	[3]

## Data Availability

Not applicable.
